# Supplementary material for: The Gini coefficient as a useful measure of malaria inequality among populations
Source: Malar J. 2020 Dec 2;19:444. doi: 10.1186/s12936-020-03489-x (PMC7709295; doi:10.1186/s12936-020-03489-x)
Supplement: Supplementary file 1 — Additional file 1: Text S1. Calculating the Gini coefficient and bootstrap 95% confidence intervals using R version 3.6.3. Table S1. Gini coefficients of inequality among WHO world regions in the estimated burden (per 100,000 of the population) of major global diseases. Table S2. No decline in the Gini coefficient of global malaria inequality based on estimated malaria cases as a percentage of Population at Risk between 2010 and 2018. Table S3. No decline in the Gini coefficient of malaria inequality among 16 West African countries based on estimated malaria cases as a percentage of Population at Risk between 2010 and 2018. Table S4. Gini coefficients show differences among West African countries in the levels of sub-national variation in malaria parasite prevalence in community surveys of children <5 years of age. Table S5. Gini coefficients show extent of variation in community prevalence of P. falciparum among 16 villages in the Garki Project in northern Nigeria in eight cross-sectional surveys during the pre-intervention phase of the project. Figure S1. High Gini coefficients of variation in P. falciparum prevalence among villages in The Gambia with seasonal malaria transmission. Variation was analysed among 20 villages each surveyed at two seasonal times. [file 12936_2020_3489_MOESM1_ESM.docx]

**Additional file 1**

**Additional file 1: Text S1.** Calculating the Gini coefficient and bootstrap 95% confidence intervals using R version 3.6.3

1. Download and start R Version 3.6.3
2. Download ***reldist*** from the R library
3. Download **boot** from the R library
4. Command: **x <- c(n1,n2,n3,n4…)**
5. Command: **gini(x)** will give an output of the gini coefficient**
6. Command: **y <- boot(x,gini,500)** to set resampling of 500
7. Command: **quantile(y$t, probs=c(0.025,0.975))**

Values of the Gini coefficient without the confidence intervals were also checked and confirmed using STATA version 15.3 and Microsoft Excel.

**Additional file 1: Table S1.** Gini coefficients of inequality among WHO world regions in the estimated burden (per 100,000 of the population) of major global diseases.

| **WHO World Region** | **Malaria 2018** | **HIV 2018** | **TB**  **2018** | **Hep C**  **2015** | **Cancer**  **2018** | **Diabetes**  **2014** | **Respiratory**  **Deaths 2016** | **CVD Deaths 2016** |
| --- | --- | --- | --- | --- | --- | --- | --- | --- |
| Africa | 23000 | 107 | 27 | 31 | 75.4 | 7100 | 18.1 | 112.8 |
| Americas | 670 | 16 | 11 | 6.4 | 373.2 | 8300 | 50.1 | 195.1 |
| East Med | 1000 | 7 | 1.3 | 62.5 | 97.4 | 13700 | 24.1 | 198.2 |
| Europe | 0.00 | 19 | 12 | 61.8 | 495.6 | 7300 | 48.9 | 435.6 |
| SE Asia | 490 | 9 | 3.5 | 14.8 | 100.7 | 8600 | 68.4 | 201.8 |
| W Pacific | 260 | 6.00 | 1.8 | 6.0 | 321.5 | 8400 | 62.2 | 290.0 |
| Gini Coefficient (95% CI) | 0.77 (0.66, 0.81) | 0.56 (0.41, 0.58) | 0.49 (0.37, 0.56) | 0.43  (0.31, 0.49) | 0.36 (0.26, 0.40) | 0.12 (0.062, 0.15) | 0.23  (0.14,  0.28) | 0.22  (0.15,  0.26) |

The Gini coefficients and 95% confidence intervals tabulated here are plotted in Figure 1. TB, tuberculosis; CVD, cardiovascular disease. Data are estimates as presented in WHO world reports and fact sheets, based on the most recent available, with references given in the paper.

**Additional file 1: Table S2.** No decline in the Gini coefficient of global malaria inequality based on estimated malaria cases as a percentage of Population at Risk between 2010 and 2018.

| **WHO World Region** | **2010** | **2011** | **2012** | **2013** | **2014** | **2015** | **2016** | **2017** | **2018** |
| --- | --- | --- | --- | --- | --- | --- | --- | --- | --- |
| Africa | 29.5 | 27.9 | 26.6 | 25.3 | 23.8 | 23.3 | 23.5 | 23.5 | 23.0 |
| Americas | 0.65 | 0.48 | 0.45 | 0.43 | 0.36 | 0.42 | 0.51 | 0.69 | 0.67 |
| East Med | 1.0 | 1.1 | 0.96 | 0.88 | 0.88 | 0.82 | 1.0 | 1.0 | 1.0 |
| European | 5x10^-4^ | 2x10^-4^ | 5x10^-4^ | 7x10^-6^ | 5x10^-6^ | 0.0 | 0.0 | 0.0 | 0.0 |
| SE Asia | 1.7 | 1.4 | 1.2 | 0.89 | 0.85 | 0.87 | 0.88 | 0.71 | 0.49 |
| W Pacific | 0.26 | 0.22 | 0.24 | 0.28 | 0.32 | 0.19 | 0.23 | 0.24 | 0.26 |
| Gini Coefficient  (95% CI) | 0.78  (0.67,  0.82) | 0.77  (0.66, 0.81) | 0.77  (0.66, 0.81) | 0.77  (0.66, 0.81) | 0.77  (0.66, 0.81) | 0.77  (0.66, 0.81) | 0.77  (0.66, 0.81) | 0.76  (0.65, 0.80) | 0.77 (0.66, 0.81) |

Data are estimates of numbers of cases and populations at risk as presented in the WHO World Malaria Report 2019.

**Additional file 1: Table S3.** No decline in the Gini coefficient of malaria inequality among 16 West African countries based on estimated malaria cases as a percentage of Population at Risk between 2010 and 2018.

| **Country** | **2010** | **2011** | **2012** | **2013** | **2014** | **2015** | **2016** | **2017** | **2018** |
| --- | --- | --- | --- | --- | --- | --- | --- | --- | --- |
| Benin | 38.8 | 37.0 | 37.8 | 39.5 | 39.9 | 41.2 | 42.2 | 39.9 | 38.6 |
| Burkina Faso | 55.1 | 54.0 | 52.8 | 48.8 | 43.6 | 40.0 | 40.2 | 40.0 | 39.9 |
| Cape Verde | 0.04 | 0.01 | 0.0008 | 0.016 | 0.019 | 0.01 | 0.035 | 0.30 | 0.00 |
| The Gambia | 28.9 | 25.7 | 27.5 | 23.7 | 14.2 | 19.5 | 11.65 | 5.30 | 6.60 |
| Ghana | 36.4 | 38.0 | 37.43 | 34.93 | 31.58 | 27.7 | 23.60 | 21.3 | 22.4 |
| Guinea | 41.5 | 42.7 | 42.8 | 40.8 | 38.1 | 35.6 | 33.1 | 31.2 | 28.4 |
| Guinea-Bissau | 13.4 | 14.1 | 12.9 | 11.3 | 9.4 | 8.0 | 7.1 | 7.8 | 12.3 |
| Ivory Coast | 46.9 | 44.2 | 39.6 | 33.9 | 31.5 | 32.0 | 35.5 | 36.2 | 33.1 |
| Liberia | 34.6 | 33.0 | 30.8 | 31.7 | 33.8 | 34.7 | 38.6 | 40.1 | 36.2 |
| Mali | 38.4 | 40.5 | 43.6 | 45.3 | 44.1 | 39.2 | 38.4 | 38.7 | 38.7 |
| Mauritania | 3.9 | 4.8 | 2.8 | 3.3 | 4.9 | 6.2 | 7.2 | 5.5 | 3.9 |
| Niger | 42.6 | 42.8 | 43.1 | 42.0 | 40.0 | 37.0 | 35.9 | 35.7 | 35.7 |
| Nigeria | 39.9 | 37.3 | 34.8 | 32.9 | 31.4 | 29.6 | 28.1 | 28.3 | 29.2 |
| Senegal | 5.9 | 5.0 | 5.7 | 6.8 | 4.0 | 7.0 | 4.6 | 5.2 | 5.6 |
| Sierra Leone | 45.9 | 45.4 | 44.7 | 43.3 | 40.9 | 40.4 | 39.1 | 36.6 | 32.0 |
| Togo | 30.9 | 31.3 | 35.0 | 38.5 | 38.5 | 36.4 | 32.5 | 27.8 | 26.7 |
| Gini Coefficient  (95% CI) | 0.28  (0.19, 0.36) | 0.28  (0.19, 0.36) | 0.28  (0.17, 0.36) | 0.28  (0.20, 0.38) | 0.29  (0.18, 0.36) | 0.27  (0.21, 0.37) | 0.30  (0.21, 0.37) | 0.32  (0.22, 0.36) | 0.31  (0.22, 0.39) |

The Gini coefficients and 95% confidence intervals tabulated here are plotted in Figure 2. Data are estimates of numbers of cases and populations at risk as presented in the WHO World Malaria Report 2019.

**Additional file 1: Table S4.** Gini coefficients show differences among West African countries in the levels of sub-national variation in malaria parasite prevalence in community surveys of children <5 years of age

| **Prevalence in the major administrative units surveyed within each of the countries:** | | | | | | | |
| --- | --- | --- | --- | --- | --- | --- | --- |
| **Nigeria** | | **Ghana** | | **Burkina Faso** | | **Sierra Leone** | |
| Benue | 44.5 | Western | 23.5 | Boucle | 23.3 | Kailahun | 45 |
| Kogi | 5.4 | Central | 30.2 | Cascades | 13 | Kenema | 37.7 |
| Kwara | 26.4 | Accra | 4.8 | Centre | 7.2 | Kono | 37.5 |
| Nasarawa | 35.9 | Volta | 27.5 | E Centre | 12.4 | Bombali | 37.6 |
| Niger | 33.5 | Eastern | 31.3 | N Centre | 18.2 | Kambia | 48.3 |
| Plateau | 35.8 | Ashanti | 16.6 | W Centre | 25.4 | Koinadugu | 57.9 |
| Adamawa | 34.7 | Brong Ahafo | 22.4 | S Centre | 12.3 | Port Loko | 58.5 |
| Bauchi | 19.6 | Northern | 25.2 | East | 17.6 | Tonkolili | 55.7 |
| Gombe | 28.6 | Upper East | 14.7 | Hauts-Bas | 12.7 | Bo | 39.7 |
| Taraba | 42.9 | Upper West | 21.5 | North | 11.4 | Bonthe | 26.1 |
| Yobe | 18.9 |  |  | Plateau C | 8.4 | Moyamba | 39.9 |
| Jigawa | 27.9 |  |  | Sahel | 18.2 | Pujehun | 46.8 |
| Kaduna | 36.7 |  |  | South-West | 38.9 | W Rural | 34.9 |
| Oyo | 19.2 |  |  |  |  | W Urban | 6.3 |
| Abia | 8.2 |  |  |  |  |  |  |
| Anambra | 10.2 |  |  |  |  |  |  |
| Ebonyi | 30 |  |  |  |  |  |  |
| Enugu | 10.5 |  |  |  |  |  |  |
| Imo | 5.1 |  |  |  |  |  |  |
| Akwa Ibom | 22.8 |  |  |  |  |  |  |
| Bayelsa | 31.4 |  |  |  |  |  |  |
| Cross River | 26.1 |  |  |  |  |  |  |
| Delta | 20.4 |  |  |  |  |  |  |
| Edo | 18.6 |  |  |  |  |  |  |
| Rivers | 7.3 |  |  |  |  |  |  |
| Ekiti | 28.8 |  |  |  |  |  |  |
| Lagos | 0.0 |  |  |  |  |  |  |
| Osun | 33.4 |  |  |  |  |  |  |
| Kano | 27.7 |  |  |  |  |  |  |
| Katsina | 27.8 |  |  |  |  |  |  |
| Kebbi | 63.6 |  |  |  |  |  |  |
| Sokoto | 46.6 |  |  |  |  |  |  |
| Zamfara | 62.6 |  |  |  |  |  |  |
| Ogun | 14.7 |  |  |  |  |  |  |
| Ondo | 21.3 |  |  |  |  |  |  |
| Gini  (95% CI) | 0.30  (0.26, 0.35) | | 0.19  (0.12, 0.25) | | 0.25  (0.19, 0.29) | | 0.17  (0.12,0.22) |

The Gini coefficients and 95% confidence intervals tabulated here are plotted in Figure 3. Data are from national Multiple Indicator Surveys with references in the paper.

**Additional file 1: Table S5.** Gini coefficients show extent of variation in community prevalence of *P. falciparum* among 16 villages in the Garki Project in northern Nigeria in eight cross-sectional surveys during the pre-intervention phase of the project.

| **Village** | **Percent prevalence at each of eight surveys** | | | | | | | |
| --- | --- | --- | --- | --- | --- | --- | --- | --- |
|  | 1 | 2 | 3 | 4 | 5 | 6 | 7 | 8 |
| Mukawa | 54.3 | 48.0 | 27.4 | 39.4 | 36.6 | 37.8 | 39.2 | 38.0 |
| Matsari | 63.6 | 41.3 | 34.6 | 39.3 | 41.9 | 46.5 | 30.0 | 35.6 |
| Rafin Marke | 57.8 | 32.2 | 44.1 | 43.6 | 58.4 | 47.6 | 33.6 | 37.1 |
| Kukar | 66.6 | 40.0 | 36.2 | 40.3 | 57.7 | 45.4 | 30.2 | 41.9 |
| UngwarBako | 64.1 | 39.3 | 35.5 | 40.8 | 49.0 | 50.2 | 37.0 | 37.6 |
| Kargo Kudu | 50.1 | 24.7 | 32.0 | 42.1 | 47.6 | 41.0 | 23.5 | 30.3 |
| Nasakar | 62.5 | 38.8 | 42.4 | 43.7 | 60.5 | 51.2 | 20.7 | 37.1 |
| Jaya | 57.3 | 40.0 | 22.1 | 33.4 | 39.1 | 46.1 | 32.1 | 21.3 |
| Sugungun | 54.4 | 47.0 | 41.2 | 42.9 | 53.3 | 55.6 | 47.6 | 44.8 |
| Karama | 55.8 | 49.6 | 43.0 | 38.6 | 55.1 | 55.5 | 43.2 | 39.7 |
| Batakashi | 60.2 | 44.2 | 50.0 | 49.0 | 50.0 | 67.1 | 39.6 | 43.2 |
| Nabanawa | 63.2 | 41.0 | 40.8 | 44.0 | 58.6 | 46.5 | 34.1 | 38.4 |
| Ajura | 49.7 | 33.5 | 37.6 | 38.9 | 56.7 | 44.2 | 32.4 | 34.7 |
| Barebari | 60.8 | 50.6 | 47.6 | 46 | 53.4 | 61.5 | 42.3 | 39.5 |
| Kwaru | 55.6 | 39.6 | 46.4 | 45.9 | 54.1 | 63.4 | 49.5 | 38.5 |
| Tafin Sale | 45.7 | 26.9 | 31.4 | 35.3 | 40.0 | 39.8 | 35.5 | 37.3 |
| Gini Coefficient (95% CI) | 0.056  (0.043, 0.062) | 0.10  (0.075,  0.12) | 0.11  (0.088,  0.13) | 0.053  (0.042,  0.058) | 0.082  (0.059,  0.09) | 0.093  (0.077,  0.11) | 0.17  (0.11, 0.20) | 0.070  (0.044,  0.09) |

The Gini coefficients and data tabulated here are plotted in Figure 4. Each of the surveys was approximately 10 weeks apart, and variation among the villages was highest in the dry season.


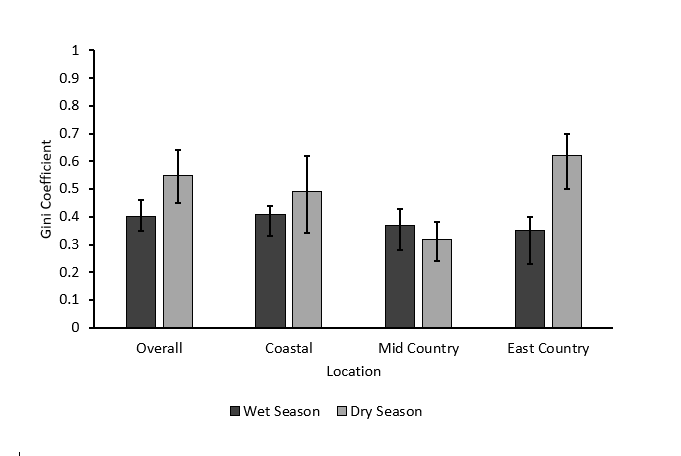


**Additional file 1: Figure S1. High Gini coefficients of variation in *P. falciparum* prevalence among villages in The Gambia with seasonal malaria transmission**. Variation was analysed among 20 villages each surveyed at two seasonal times. There were 3860 individuals sampled in the wet season and 3707 in the dry season (prevalence measurements in each of the villages are previously published). The coastal area had 6 villages sampled, while the mid country area and east country areas each had 7 villages sampled. The east country area showed statistically greater variation among villages in the dry season.
